# Visual Sensing and Depth Perception for Welding Robots and Their Industrial Applications

**DOI:** 10.3390/s23249700

**Published:** 2023-12-08

**Authors:** Ji Wang, Leijun Li, Peiquan Xu

**Affiliations:** 1 Department of Chemical and Materials Engineering, University of Alberta, Edmonton, AB T6G 1H9, Canada; 2 School of Materials Science and Engineering, Shanghai University of Engineering Science, Shanghai 201620, China

**Keywords:** welding sensor, welding robot, depth perception, 3D reconstruction, deep learning, industrial applications

## Abstract

With the rapid development of vision sensing, artificial intelligence, and robotics technology, one of the challenges we face is installing more advanced vision sensors on welding robots to achieve intelligent welding manufacturing and obtain high-quality welding components. Depth perception is one of the bottlenecks in the development of welding sensors. This review provides an assessment of active and passive sensing methods for depth perception and classifies and elaborates on the depth perception mechanisms based on monocular vision, binocular vision, and multi-view vision. It explores the principles and means of using deep learning for depth perception in robotic welding processes. Further, the application of welding robot visual perception in different industrial scenarios is summarized. Finally, the problems and countermeasures of welding robot visual perception technology are analyzed, and developments for the future are proposed. This review has analyzed a total of 2662 articles and cited 152 as references. The potential future research topics are suggested to include deep learning for object detection and recognition, transfer deep learning for welding robot adaptation, developing multi-modal sensor fusion, integrating models and hardware, and performing a comprehensive requirement analysis and system evaluation in collaboration with welding experts to design a multi-modal sensor fusion architecture.

## 1. Introduction

**Figure 1:**
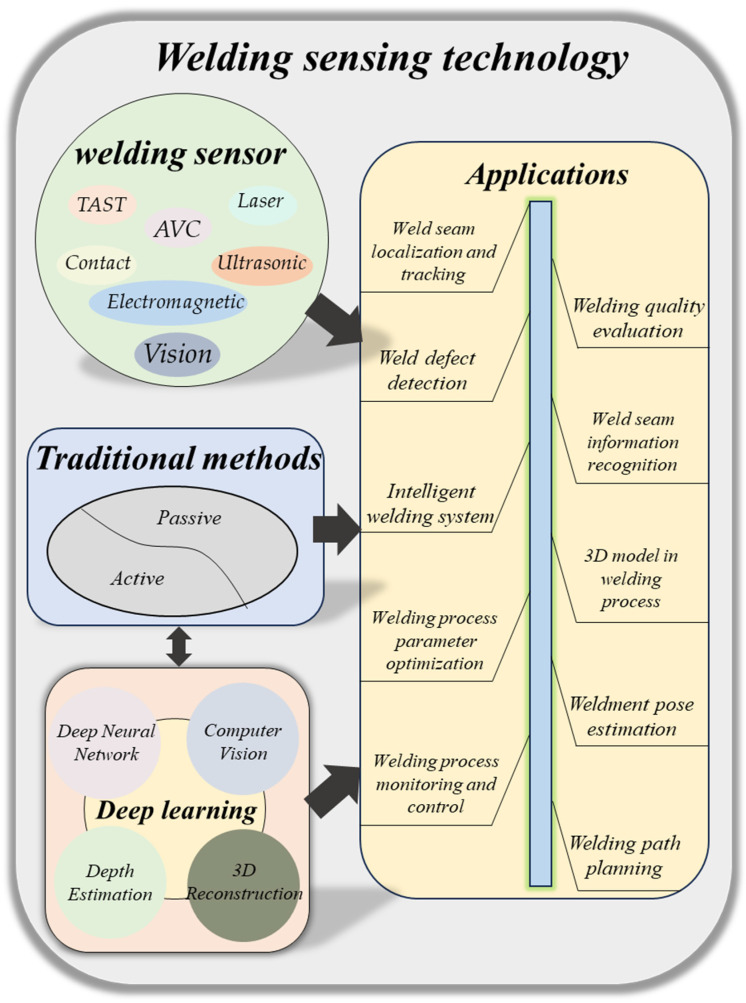
A classification of depth perception for welding robots.

The interaction between cameras and welding lies in the integration of technology, vision, and field plots for controlling the welding process [1,2]. As we embrace the rapid development of artificial intelligence [3], the prospects for research and development in the automation and intelligence of robotic welding have never been more promising [4,5,6]. Scientists, engineers, and welders have been exploring new methods for automated welding. Over the past few decades, as shown in Figure 1, numerous sensors have been developed for welding, including infrared sensors [7], vision sensors [8,9], temperature sensors [10], acoustic sensors [11], arc sensors [12], and force sensors [13].

The vision sensor stands out as one of the sensors with immense development potential. This device leverages optical principles and employs image processing algorithms to capture images while distinguishing foreground objects from the background. Essentially, it amalgamates the functionalities of a camera with sophisticated image processing algorithms to extract valuable signals from images [14].

Vision sensors find widespread application in industrial automation and robotics, serving various purposes including inspection, measurement, object detection, quality control, and navigation [15]. These versatile tools are employed across industries such as manufacturing, food safety [16], automotives, electronics, pharmaceuticals, logistics, and unmanned aerial vehicles [17]. Their utilization significantly enhances efficiency, accuracy, and productivity by automating visual inspection and control processes.

A vision sensor may also include other features such as lighting systems to enhance image quality, communication interfaces for data exchange, and integration with control systems or robots. It works in a variety of lighting conditions for detecting complex patterns, colors, shapes, and textures. Vision sensors can process visual information in real time, allowing automated systems to make decisions and take actions.

Vision sensors for welding have the characteristics of non-contact measurement, versatility, high precision, and real-time sensing [18], providing powerful information for the automated control of welding [19]. However, extracting depth information is challenging in the application of vision sensors. Depth perception is the ability to perceive the three-dimensional (3D) world through measuring the distance to objects [20,21] by using a visual system [22,23,24] mimicking human stereoscopic vision and the accommodative mechanism of the human eye [25,26,27,28]. Depth perception has a wide range of applications [29,30], such as intelligent robots [31,32], facial recognition [33,34], medical imaging [35], food delivery robots [36], intelligent healthcare [37], autonomous driving [38], virtual reality and augmented reality [39], object detection and tracking [40], human–computer interaction [41], 3D reconstruction [42], and welding robots [43,44,45].

The goal of this review is to summarize and interpret the research in depth perception and its application to welding vision sensors and evaluate some examples of robotic welding based on vision sensors.

Review [46] focuses on structured light sensors for intelligent welding robots. Review [47] focuses on vision-aided robotic welding, including the detection of various groove and joint types using active and passive visual sensing methods. Review [48] focuses on visual perception for different forms of industry intelligence. Review [49] focuses on deep learning methods for vision systems intended for Construction 4.0. The difference our review provides is a comprehensive analysis of visual sensing and depth perception. We contribute to visual sensor technology, welding robot sensors, computer vision-based depth perception methods, and the industrial applications of perception to welding robots.

## 2. Research Method

This article focuses on visual sensing and depth perception for welding robots, as well as the industrial applications. We conducted a literature review and evaluated from several perspectives, including welding robot sensors, machine vision-based depth perception methods, and the welding robot sensors used in industry.

**Figure 2:**
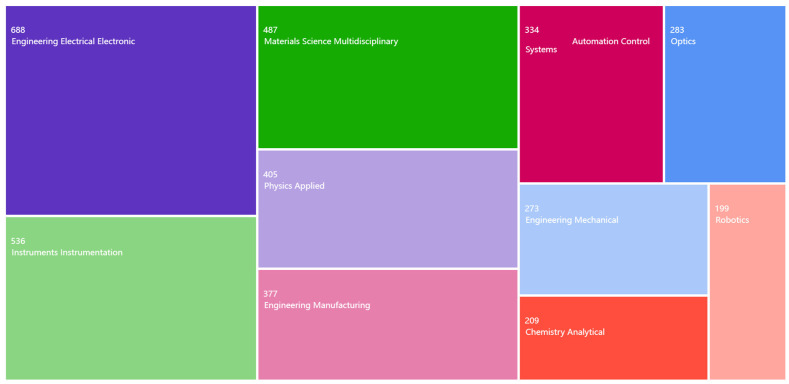
Top ten fields and the number of papers in each field. The number of retrieved papers was 2662.

We searched for relevant literature in the Web of Science database using the search term “Welding Sensors”. A total of 2662 articles were retrieved. As shown in Figure 2, these articles were categorized into subfields and the top 10 fields, and their respective number of articles were plotted. From each subfield, we selected representative articles and reviewed them further. Valuable references from their bibliographies were subsequently collected.

In total, we selected 152 articles as references for this review. Our criterion for literature selection was the quality of the articles, specifically focusing on the following:

1. Relevance to technologies of visual sensors for welding robots.
2. Sensors used in the welding process.
3. Depth perception methods based on computer vision.
4. Welding robot sensors used in industry.

## 3. Sensors for Welding Process

**Figure 3:**
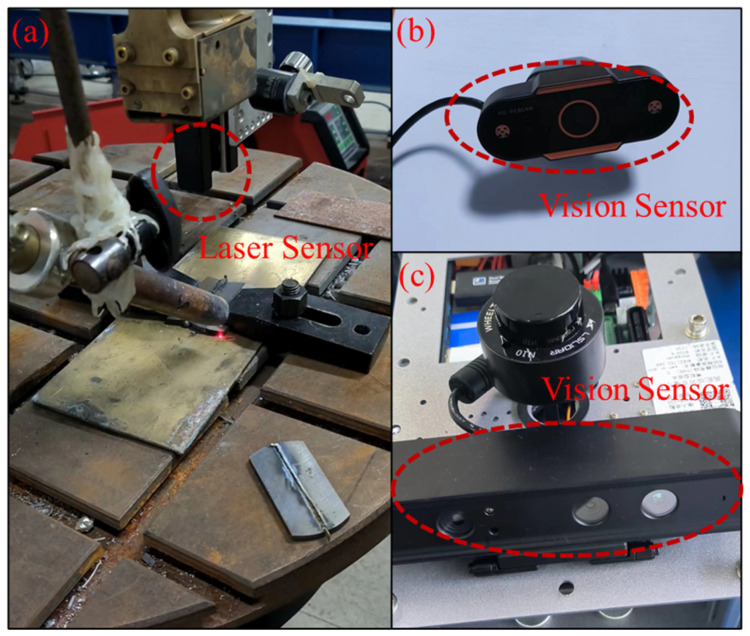
(**a**) A typical laser vison sensor setup for arc welding process; (**b**) a video camera as a vision sensor; (**c**) a vision sensor with multiple lenses.

Figure 3 shows a typical laser vison sensor used for a welding process. If there are changes in the joint positions, the sensors used for searching the welding seam will provide real-time information to the robot controller. Commonly used welding sensors include thru-arc seam tracking (TAST) sensors, arc voltage control (AVC) sensors, touch sensors, electromagnetic sensors, supersonic sensors, laser vision sensors, etc.

### 3.1. Thru-Arc Seam Tracking (TAST) Sensors

In 1990, Siores [50] achieved weld seam tracking and the control of weld pool geometry using the arc as a sensor. The signal detection point is the welding arc, eliminating sensor positioning errors and being unaffected by arc spatter, smoke, or arc glare, making it a cost-effective solution. Comprehensive mathematical models [51,52] have been developed and successfully applied to automatic weld seam tracking in arc welding robots and automated welding equipment. Commercial robot companies have equipped their robots such sensing devices [53].

Arc sensor weld seam tracking utilizes the arc as a sensor to detect changes in the welding current caused by variations in the arc length [54]. The sensing principle is because when the arc position changes, the electrical parameters of the arc also change, primarily in the distance between the welding nozzle and the surface of the workpiece. From this, the relative position deviation between the welding gun and the weld seam can be derived from the arc oscillation pattern. In many cases, the typical thru-arc seam tracking (TAST) control method can optimize the weld seam tracking performance by adjusting various variables.

The advantages of TAST as a weld seam tracking method are its low cost, as it only requires a welding current sensor as hardware. However, it requires the construction of a weld seam tracking control model, where the robot adjusts the torch position in response to the welding current feedback.

### 3.2. Arc Voltage Control (AVC) Sensors

In gas tungsten arc welding (GTAW), there is a proportional relationship between the arc voltage and arc length. AVC sensors are used to monitor changes in the arc voltage when there are variations in the arc length, providing feedback to control the torch height [55]. Due to their lower sensitivity to arc length signals, AVC sensors are primarily used for vertical tracking, and, less frequently, are used for horizontal weld seam tracking. The establishment of an AVC sensing model is relatively simple and can be used in both pulsed current welding and constant current welding.

### 3.3. Laser Sensors

Due to material or process limitations, certain welding processes, such as thin plate welding, cannot utilize arc sensors for weld seam tracking. Additional sensors on the robotic system are required; a popular choice are laser sensors.

Laser sensors do not require an arc model and can determine the welding joint position before welding begins. When there are changes in the joint, the robot dynamically adjusts the welding parameters or corrects the welding path deviations in real time [56]. Laser sensor systems are relatively complex and have stringent requirements for the welding environment. Since the laser sensor is installed on the welding torch, it may limit the accessibility of the torch to the welding joint. An associated issue is that it introduces the inconsistency between the position of the laser sensor’s detection point and the welding point, known as sensor positioning lead error.

### 3.4. Contact Sensing

Contact sensors do not require any weld seam tracking control functions. Instead, they find the weld seam before initiating the arc and continuously adjust the position deviation along the entire path. The robot operates in a search mode, using contact to gather the three-dimensional positional information of the weld seam. The compensation for the detected deviation is then transmitted to the robot controller.

Typical contact-based weld seam tracking sensors rely on probes that roll or slide within the groove to reflect the positional deviation between the welding torch and the weld seam [57]. They utilize microswitches installed within the sensor to determine the polarity of the deviation, enabling weld seam tracking. Contact sensors are suitable for X-and Y-shaped grooves, narrow gap welds, and fillet welds. Contact sensors are widely used in seam tracking, because of their simple system structure, easy operation, low cost, and the fact they are not affected by arc smoke or spatter. However, they have some drawbacks, including different groove types requiring different probes, and the probes potentially experiencing significant wear and deform easily, which are not suitable for high-speed welding processes.

### 3.5. Ultrasonic Sensing

The detection principle of ultrasonic weld seam tracking sensors is as follows: Ultrasonic waves are emitted by the sensor and when they reach the surface of the welded workpiece, they are reflected and received by the ultrasonic sensor. By calculating the time interval between the emission and reception of the ultrasonic waves, the distance between the sensor and the workpiece can be determined. For weld seam tracking, the edge-finding method is used to detect the left and right edge deviations of the weld seam. Ultrasonic sensing can be applied in welding methods such as GTAW welding and submerged arc welding (SAW) and enable the automatic recognition of the welding workpiece [58,59]. Ultrasonic sensing offers significant advantages in the field of welding, including non-contact measurement, high precision, real-time monitoring, and wide frequency adaptability. By eliminating interference with the welding workpiece and reducing sensor wear, it ensures the accuracy and consistency of weld joints. Furthermore, ultrasonic sensors enable the prompt detection of issues and defects, empowering operators to take timely actions and ensure welding quality. However, there are limitations to ultrasonic sensing, such as high costs, stringent environmental requirements, material restrictions, near-field detection sensitivity, and operational complexities. Therefore, when implementing ultrasonic sensing, a comprehensive assessment of specific requirements, costs, and technological considerations is essential.

### 3.6. Electromagnetic Sensing

Electromagnetic sensors utilize the changes in induced currents in sensing coils caused by variations in the induced currents in the surrounding metal near the sensor. This allows the sensor to perceive the position deviations for the welding joint. Dual electromagnetic sensors can detect the offset of the weld seam from the center position of the sensor [60,61]. They are particularly suitable for butt welding processes of structural profiles, especially for detecting position deviations in welding joints with painted surfaces, markings, and scratches. They can also achieve the automatic recognition of gapless welding joint positions. Kim et al. [62] developed dual electromagnetic sensors for the arc welding process of I-shaped butt joints in structural welding. They performed weld seam tracking by continuously correcting the offset of the sensor’s position in real time.

### 3.7. Vision Sensor

Vision sensing systems can be divided into active vision sensors and passive vision sensors according to the imaging light source in the vision system. Passive vision sensors are mainly used for extracting welding pool information, analyzing the transfer of molten droplets, recognizing weld seam shapes, and weld seam tracking. In [63], a passive optical image sensing system with secondary filtering capability for the intelligent extraction of aluminum alloy welding pool images was proposed based on spectral analysis, which obtained clear images of aluminum alloy welding pools.

Active vision sensors utilize additional imaging light sources, typically lasers. The principle is to use a laser diode and a CCD camera to form a vision sensor. The red light emitted by the laser diode is reflected in the welding area and enters the CCD camera. The relative position of the laser beam in the image is used to determine the three-dimensional information of the weld seam [64,65,66]. To prevent interference from the complex spectral composition of the welding arc, and to improve the imaging quality, specific wavelength lasers can be used to isolate the arc light. Depth calculation methods include Fourier transform, phase measurement, Moiré contouring, and optical triangulation. Essentially, they analyze the spatial light field modulated by the surface of the object to obtain the three-dimensional information of the welded workpiece.

Both passive and active vision sensing systems can achieve two-dimensional or three-dimensional vision for welding control. Two-dimensional sensing is mainly used for weld seam shape recognition and monitoring of the welding pool. Three-dimensional sensing can construct models of important depth information for machine vision [67,68].

## 4. Depth Perception Method Based on Computer Vision

**Figure 4:**
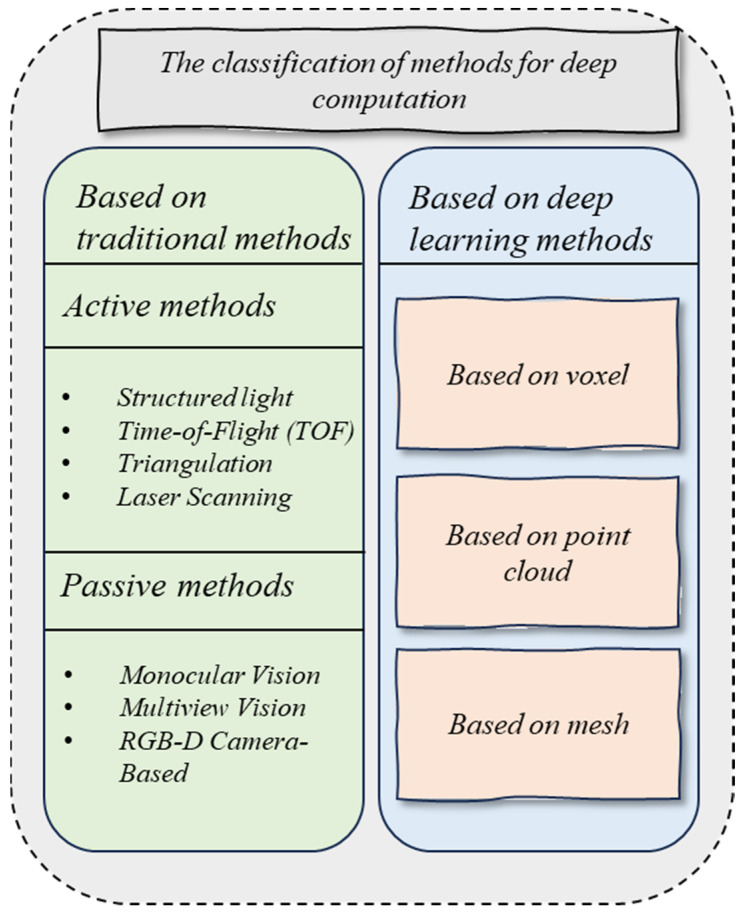
A classification of deep computation, which can be broadly divided into traditional methods and deep learning methods, is shown.

Currently, 3D reconstruction has been widely applied in robotics [69], localization and navigation [70], and industrial manufacturing [71]. Figure 4 illustrates the two categories of methods for deep computation. The traditional 3D reconstruction algorithms are based on multi-view geometries. These algorithms utilize image or video data captured from multiple viewpoints and employ geometric calculations and disparity analysis to reconstruct the geometric shape and depth information of objects in the 3D space. Methods based on multi-view geometry typically involve camera calibration, image matching, triangulation, and voxel filling steps to achieve high-quality 3D reconstructions.

**Figure 5:**
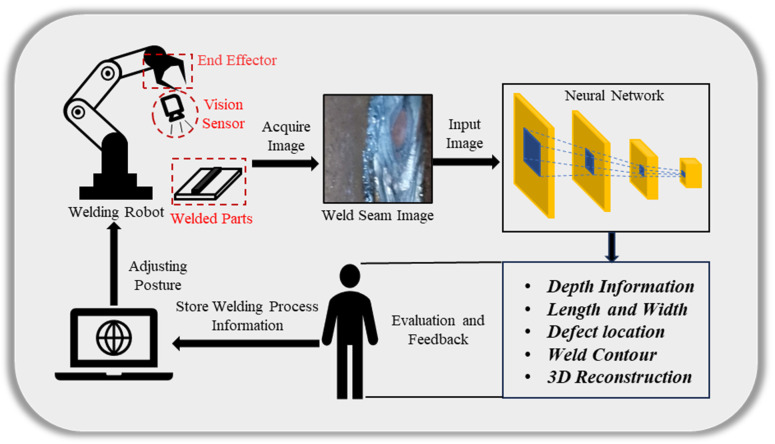
A schematic of the processing sequence of welding robot vision perception. The welding robot obtains the welding images from the vision sensor, processes various welding information through the neural network, and then evaluates and feeds back to correct the welding operation and improves the accuracy.

Figure 5 describes the visual perception for welding robots based on deep learning, including 3D reconstruction. Deep learning algorithms leverage convolutional neural networks (CNNs) to tackle the problem of 3D reconstruction. By applying deep learning models to image or video data, these algorithms can acquire the 3D structure and depth information of objects through learning and inference. Through end-to-end training and automatic feature learning, these algorithms can overcome the limitations of traditional approaches and achieve better performance in 3D reconstruction.

### 4.1. Traditional Methods for 3D Reconstruction Algorithms

Traditional 3D reconstruction algorithms can be classified into two categories according to whether the sensor actively illuminates the objects or not [72]. The active methods utilize laser, sound, or electromagnetic waves to emit toward the target objects and to receive the reflected waves. The passive methods rely on cameras capturing the reflection of the ambient environment (e.g., natural light), and specific algorithms to calculate the 3D spatial information of the objects.

In the active methods, by measuring the changes in the properties of the returned light waves, sound waves, or electromagnetic waves, the depth information of the objects can be inferred. The precise calibration and synchronization of hardware devices and sensors are required to ensure the accuracy and reliability.

In contrast, for the passive methods, the captured images are processed by algorithms to obtain the objects’ 3D spatial information [73,74]. These algorithms typically involve feature extraction, matching, and triangulation to infer the depth and shape information of the objects in the images.

#### 4.1.1. Active Methods

**Figure 6:**
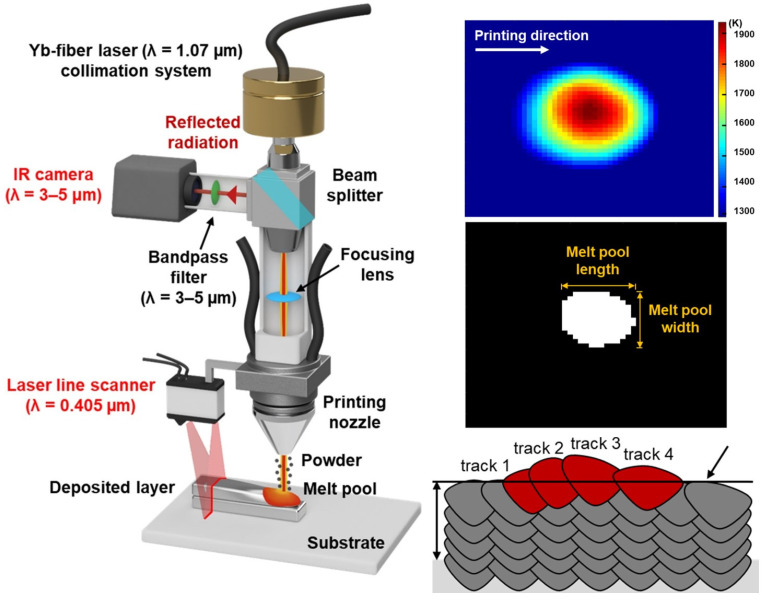
Depth perception based on laser line scanner and coaxial infrared camera for directed energy deposition (DED) process. Additional explanations for the symbols and color fields can be found in [87]. Reprinted with permission from [87].

Figure 6 shows schematic diagrams of several active methods. Table 1 summarizes the relevant literature on the active methods.

**Table 1:** Active approaches in the selected papers.

Year	Method	Description	References
2019	Structured light	A new active light field depth estimation method is proposed.	[75]
2015	Structured light	A structured light system for enhancing the surface texture of objects is proposed.	[76]
2021	Structured light	A global cost minimization framework is proposed for depth estimation using phase light field and re-formatted phase epipolar plane images.	[77]
2024	Structured light	A novel active stereo depth perception method based on adaptive structured light is proposed.	[78]
2023	Structured light	A parallel CNN transformer network is proposed to achieve an improved depth estimation for structured light images in complex scenes.	[79]
2022	Time-of-Flight (TOF)	DELTAR is proposed to enable lightweight Time-of-Flight sensors to measure high-resolution and accurate depth by collaborating with color images.	[80]
2020	Time-of-Flight (TOF)	Based on the principle and imaging characteristics of TOF cameras, a single pixel is considered as a continuous Gaussian source, and its differential entropy is proposed as an evaluation parameter.	[81]
2014	Time-of-Flight (TOF)	Time-of-Flight cameras are presented and common acquisition errors are described.	[82]
2003	Triangulation	A universal framework is proposed based on the principle of triangulation to address various depth recovery problems.	[83]
2021	Triangulation	Laser power is controlled via triangulation camera in a remote laser welding system.	[84]
2020	Triangulation	A data acquisition system is assembled based on differential laser triangulation method.	[85]
2017	Laser scanning	The accuracy of monocular depth estimation is improved by introducing 2D plane observations from the remaining laser rangefinder without any additional cost.	[86]
2021	Laser scanning	An online melt pool depth estimation technique is developed for the directed energy deposition (DED) process using a coaxial infrared (IR) camera, laser line scanner, and artificial neural network (ANN).	[87]
2018	Laser scanning	An automatic crack depth measurement method using image processing and laser methods is developed.	[88]

Structured light—a technique that utilizes a projector to project encoded structured light onto the object being captured, which is then recorded by a camera [75]. This method relies on the differences in the distance and direction between the different regions of the object relative to the camera, resulting in variations in the size and shape of the projected pattern. These variations can be captured by the camera and processed by a computational unit to convert them into depth information, thus acquiring the three-dimensional contour of the object [76]. However, structured light has some drawbacks, such as susceptibility to interference from ambient light, leading to poor performance in outdoor environments. Additionally, as the detection distance increases, the accuracy of structured light decreases. To address these issues, current research efforts have employed strategies such as increasing power and changing coding methods [77,78,79].

Time-of-Flight (TOF)—a method that utilizes continuous light pulses and measures the time or phase difference of the received light to calculate the distance to the target [80,81,82]. However, this method requires highly accurate time measurement modules to achieve sufficient ranging precision, making it relatively expensive. Nevertheless, TOF is able to measure long distances with a minimal ambient light interference. Current research efforts are focused on reducing the yield and cost of time measurement modules while improving algorithm performance. The goal is to lower the cost by improving the manufacturing process of the time measurement module and enhance the ranging performance through algorithm optimization.

Triangulation method—a distance measurement technique based on the principles of triangulation. Unlike other methods that require precise sensors, it has a lower overall cost [83,84,85]. At short distances, the triangulation method can provide high accuracy, making it widely used in consumer and commercial products such as robotic vacuum cleaners. However, the measurement error of the triangulation method is related to the measurement distance. As the measurement distance increases, the measurement error also gradually increases. This is inherent to the principles of triangulation and cannot be completely avoided.

Laser scanning method—an active visual 3D reconstruction method that utilizes the interaction between a laser beam emitted by a laser device and the target surface to obtain the object’s three-dimensional information. This method employs laser projection and laser ranging techniques to capture the position of laser points or lines and calculate their three-dimensional coordinates, enabling accurate 3D reconstruction. Laser scanning offers advantages such as high precision, adaptability to different lighting conditions, and real-time data acquisition, making it suitable for complex shape and detail reconstruction [82]. However, this method has longer scanning times for the large objects, higher equipment costs, and challenges in dealing with transparent, reflective, or multiply scattered surfaces. With further technological advancements, laser scanning holds a vast application potential in engineering, architecture, cultural heritage preservation, and other fields. However, limitations still need to be addressed, including time, cost, and adaptability to special surfaces [86,87,88].

#### 4.1.2. Passive Methods

**Figure 7:**
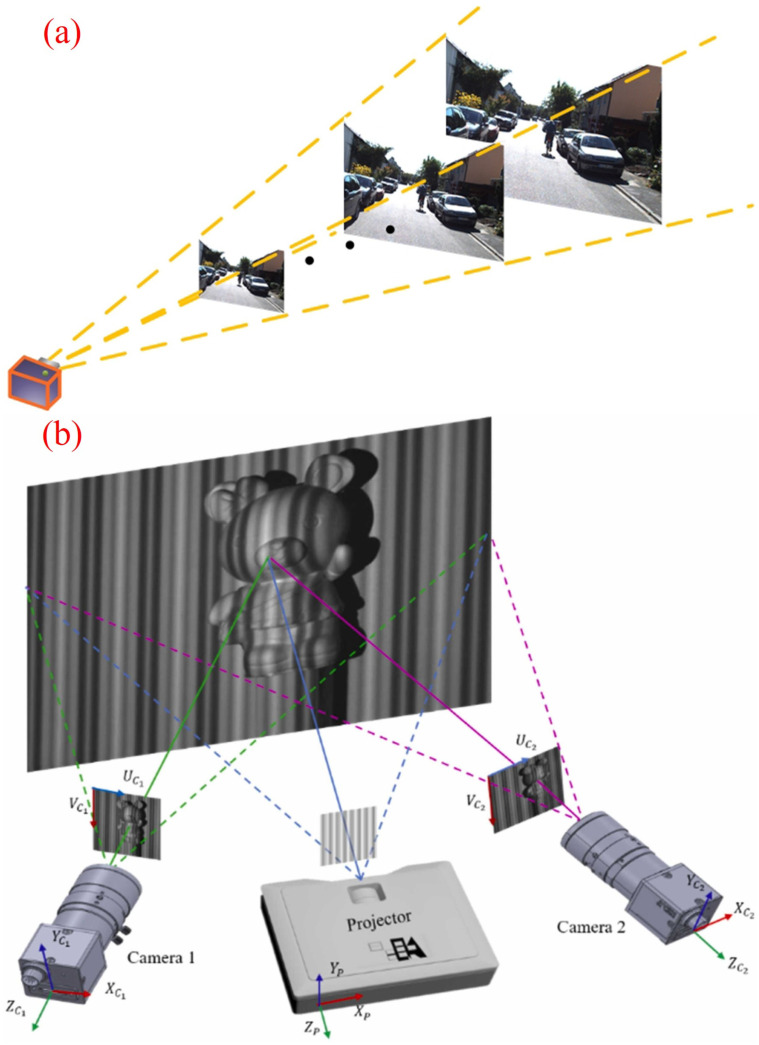
Passive depth perception methods are presented. (**a**) shows the method based on monocular vision [95]. (**b**) depicts the methods based on binocular/multi-view vision [96]. Reprinted with permission from [95,96].

Figure 7 displays schematic diagrams of several passive methods. Table 2 summarizes relevant literature on passive methods.

**Table 2:** Passive approaches in the selected papers.

Year	Method	Description	References
2010	Monocular vision	Photometric stereo	[89]
2004	Monocular vision	Shape from texture	[90]
2000	Monocular vision	Shape from shading	[91]
2018	Monocular vision	Depth from defocus	[92]
2003	Monocular vision	Concentric mosaics	[93]
2014	Monocular vision	Bayesian estimation and convex optimization techniques are combined in image processing.	[94]
2020	Monocular vision	Deep learning-based 3D position estimation	[95]
2023	Binocular/multi-view vision	Increasing the baseline distance between two cameras to improve the accuracy of a binocular vision system.	[96]
2018	Multi-view vision	Deep learning-based multi-view stereo	[97]
2020	Multi-view vision	A new sparse-to-dense coarse-to-fine framework for fast and accurate depth estimation in multi-view stereo (MVS)	[98]
2011	RGB-D camera-based	Kinect Fusion	[99]
2019	RGB-D camera-based	ReFusion	[100]
2015	RGB-D camera-based	Dynamic Fusion	[101]
2017	RGB-D camera-based	Bundle Fusion	[102]

Monocular vision—a visual depth recovery technique that uses a single camera as the capturing device. It is advantageous due to its low cost and ease of deployment. Monocular vision reconstructs the 3D environment using the disparity in a sequence of continuous images. Monocular vision depth recovery techniques include photometric stereo [89], texture recovery [90], shading recovery [91], defocus recovery [92], and concentric mosaic recovery [93]. These methods utilize variations in lighting, texture patterns, brightness gradients, focus information, and concentric mosaics to infer the depth information of objects. To improve the accuracy and stability of depth estimation, some algorithms [94,95] employ depth regularization and convolutional neural networks for monocular depth estimation. However, using monocular vision for depth estimation and 3D reconstruction has inherent challenges. A single image may correspond to multiple real-world physical scenes, making it difficult to estimate depth and achieve 3D reconstruction solely based on monocular vision methods.

Binocular/Multi-view Vision—an advanced technique based on the principles of stereo geometry. It utilizes the images captured by the left and right cameras, after rectification, to find corresponding pixels and recover the 3D structural information of the environment [96]. However, this method faces the challenge of matching the images from the left and right cameras, as inaccurate matching can significantly affect the final imaging results of the algorithm. To improve the accuracy of matching, multi-view vision introduces a configuration of three or more cameras to further enhance the precision of matching [97]. This method has notable disadvantages, including longer computation time and a poorer real-time performance [98].

RGB-D Camera-Based—in recent years, many researchers have focused on utilizing consumer-grade RGB-D cameras for 3D reconstruction. For example, Microsoft’s Kinect V1 and V2 products have made significant contributions in this area. The Kinect Fusion algorithm, proposed by Izadi et al. [99] in 2011, was a milestone in achieving real-time 3D reconstruction with RGB cameras. Subsequently, algorithms such as Dynamic Fusion [100], ReFusion [101], and Bundle Fusion [102] have emerged, further advancing the field [103]. These algorithms have provided new directions and methods using the RGB-D cameras.

### 4.2. Deep Learning-Based 3D Reconstruction Algorithms

**Figure 8:**
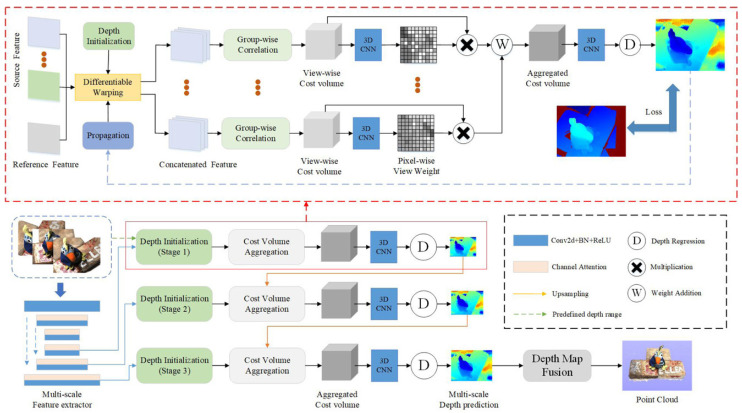
Deep learning methods based on point clouds [112]. Reprinted with permission from [112].

In the context of deep learning, image-based 3D reconstruction methods leverage large-scale data to establish prior knowledge and transform the problem of 3D reconstruction into an encoding and decoding problem. With the increasing availability of 3D datasets and improvement in computational power, deep learning 3D reconstruction methods can reconstruct the 3D models of objects from single or multiple 2D images without the need for complex camera calibration. This approach utilizes the powerful representation capabilities and data-driven learning approach of deep learning, bringing significant advancements and new possibilities to the field of image 3D reconstruction. Figure 8 illustrates schematic diagrams of several deep learning-based methods.

In 3D reconstruction, there are primarily four types of data formats: (1) The depth map is a two-dimensional image that records the distance from the viewpoint to the object for each pixel. The data is represented as a grayscale image, where darker areas correspond to closer regions. (2) Voxels are like the concept of pixels in 2D and are used to represent volume elements in 3D space. Each voxel can contain 3D coordinate information as well as other properties such as color and reflectance intensity. (3) Point clouds are composed of discrete points, where each point carries 3D coordinates and additional information such as color and reflectance intensity. (4) Meshes are two-dimensional structures composed of polygons and are used to represent the surface of 3D objects. Mesh models have the advantage of convenient computation and can undergo various geometric operations and transformations. 

The choice of an appropriate data format depends on the specific requirements and algorithm demands, providing diverse options and application areas in 3D reconstruction. Table 3 summarizes the relevant literature on deep learning-based methods. According to the different forms of processed data, we will briefly explain three types, (1) based on voxels [104,105,106,107,108], (2) based on point clouds [109,110,111,112,113,114,115], and (3) based on meshes [116,117,118,119,120,121,122].

#### 4.2.1. Voxel-Based 3D Reconstruction

Voxels are an extension of pixels to three-dimensional space and, similar to 2D pixels, voxel representations in 3D space also exhibit a regular structure. It has been demonstrated that various neural network architectures commonly used in the field of 2D image analysis can be easily extended to work for voxel representations. Therefore, when tackling problems related to 3D scene reconstruction and semantic understanding, we can leverage pixel-based representations for research. In this regard, we categorize voxel representations into dense voxel representations, sparse voxel representations, and voxel representations obtained through the conversion of point clouds. 

#### 4.2.2. Point Cloud-Based 3D Reconstruction

Traditional deep learning frameworks are built upon 2D convolutional structures, which efficiently handle regularized data structures with the support of modern parallel computing hardware. However, for images lacking depth information, especially under extreme lighting or specific optical conditions, semantic ambiguity often arises. As an extension of 3D data, 3D convolution has emerged to naturally handle regularized voxel data. However, compared to 2D images, the computational resources required for processing voxel representations grow exponentially. Additionally, 3D structures exhibit sparsity, resulting in significant resource waste when using voxel representations. Therefore, voxel representations are no longer suitable for large-scale scene analysis tasks. On the contrary, point clouds, as an irregular representation, can straightforwardly and effectively capture sparse 3D data structures, playing a crucial role in 3D scene understanding tasks. Consequently, point cloud feature extraction has become a vital step in the pipeline of 3D scene analysis and has achieved unprecedented development.

#### 4.2.3. Mesh-Based 3D Reconstruction

Mesh-based 3D reconstruction methods are techniques used for reconstructing three-dimensional shapes. This approach utilizes a mesh structure to describe the geometric shape and topological relationships of objects, enabling the accurate modeling of the objects. In mesh-based 3D reconstruction, the first step is to acquire the surface point cloud data of the object. Then, through a series of operations, the point cloud data is converted into a mesh representation. These operations include mesh topology construction, vertex position adjustment, and boundary smoothing. Finally, by optimizing and refining the mesh, an accurate and smooth 3D object model can be obtained.

Mesh-based 3D reconstruction methods offer several advantages. The mesh structure preserves the shape details of objects, resulting in higher accuracy in the reconstruction results. The adjacency relationships within the mesh provide rich information for further geometric analysis and processing. Additionally, mesh-based methods can be combined with deep learning techniques such as graph convolutional neural networks, enabling advanced 3D shape analysis and understanding.

## 5. Robotic Welding Sensors in Industrial Applications

The development of robotic welding sensors has been rapid in recent years, and their application in various industries has become increasingly widespread [123,124,125]. These sensors are designed to detect and measure various parameters such as temperature, pressure, speed, and position, which are crucial for ensuring consistent and high-quality welds. The combination of various sensors enables robotic welding machines to better perceive the welding object and control the robot to reach places that are difficult or dangerous for humans to access. As a result, robotic welding machines have been widely applied in various industries, including shipbuilding, automotive, mechanical manufacturing, aerospace, railroad, nuclear, PCB, construction, and medical equipment, due to their ability to improve the efficiency, accuracy, and safety of the welding process. Table 4 summarizes the typical applications of welding robot vision sensors in different fields.

**Figure 9:**
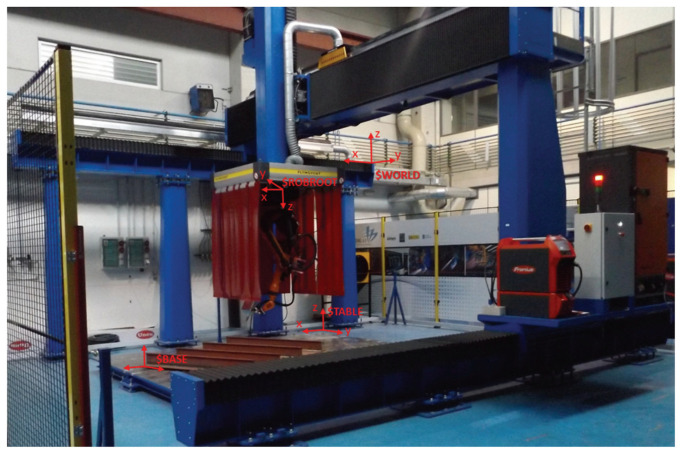
A super flexible shipbuilding welding robot unit with 9 degrees of freedom [128]. Reprinted with permission from [128].

**Figure 10:**
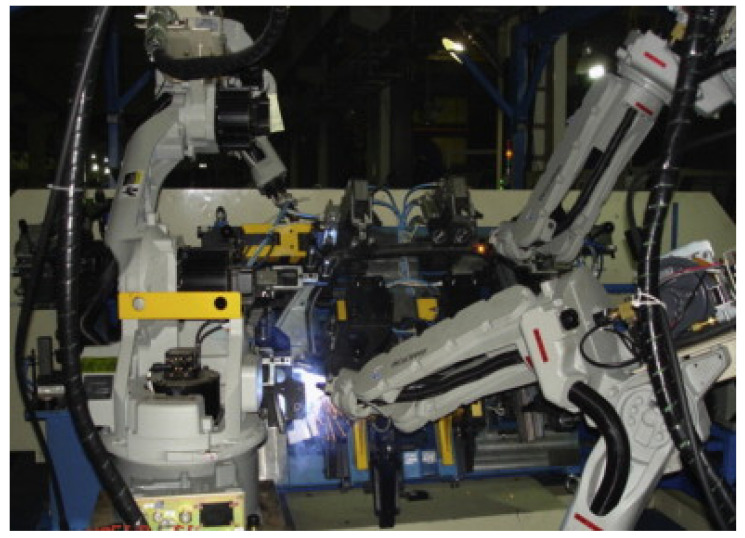
Welding robot for automobile door production [133]. Reprinted with permission from [133].

In the shipbuilding and automotive industries, robotic welding vision sensors play a crucial role in ensuring the quality and accuracy of welding processes [126,127,128,129,130,131,132,133]. These sensors are designed to detect various parameters such as the thickness and shape of steel plates, the position and orientation of car parts, and the consistency of welds. By using robotic welding vision sensors, manufacturers can improve the efficiency and accuracy of their welding processes, reduce the need for manual labor, and ensure that their products meet the required safety and quality standards. Figure 9 shows the application of welding robots in shipyards. Figure 10 shows the application of welding robots in automobile factories.

In other fields, robotic welding vision sensors can easily address complex, difficult-to-reach, and hazardous welding scenarios through visual perception [134,135,136,137,138,139,140,141,142,143,144,145,146,147,148,149]. By accurately detecting, recognizing, and modeling the object to be welded, the sensors can comprehensively grasp the structure, spatial relationships, and positioning of the object, facilitating the precise control of the welding torch and ensuring optimal welding results. The versatility of robotic welding vision sensors enables them to adapt to various environmental conditions, such as changing lighting conditions, temperatures, and distances. They can also be integrated with other sensors and systems to enhance their performance and functionality.

The use of robotic welding vision sensors offers several advantages over traditional manual inspection methods. Firstly, they can detect defects and inconsistencies in real time, allowing for immediate corrective action to be taken, which reduces the likelihood of defects and improves the overall quality of the welds. Secondly, they can inspect areas that are difficult or impossible for human inspectors to access, such as the inside of pipes or the underside of car bodies, ensuring that all welds meet the required standards, regardless of their location. Furthermore, robotic welding vision sensors can inspect welds at a faster rate than manual inspection methods, allowing for increased productivity and efficiency [150]. They also reduce the need for manual labor, which can be time-consuming and costly. Additionally, the use of robotic welding vision sensors can help to improve worker safety by reducing the need for workers to work in hazardous environments [151].

We have analyzed the experimental results from the literature in actual work environments. In reference [144], the weighted function of the position error in the image space transitioned from 0 to 1, and after active control, the manipulation error was reduced to less than 2 pixels. Reference [147] utilized tool path adaptation and adaptive strategies in a robotic system to compensate for inaccuracies caused by the welding process. Experiments have demonstrated that robotic systems can operate within a certain range of outward angles, in addition to multiple approach angles of up to 50 degrees. This adaptive technique has enhanced the existing structures and repair technologies through incremental spot welding.

In summary, robotic welding vision sensors play a crucial role in assisting robotic welding systems to accurately detect and recognize the objects to be welded, and then guide the welding process to ensure optimal results. These sensors utilize advanced visual technologies such as cameras, lasers, and computer algorithms to detect and analyze the object’s shape, size, material, and other relevant features. They can be integrated into the robotic welding system in various ways, such as mounting them on the robot’s arm or integrating them into the welding torch itself. The sensors provide real-time information to the robotic system, enabling it to adjust welding parameters such as speed, pressure, and heat input to optimize weld quality and consistency [152]. Customized approaches are crucial when applying welding robots across different industries. The automotive, aerospace, and shipbuilding sectors face unique welding challenges that require tailored solutions. Customized robot designs, specialized parameters, and quality control should be considered to ensure industry-specific needs are met.

## 6. Existing Issues, Proposed Solutions, and Possible Future Work

Visual perception in welding robots encounters a myriad of challenges, encompassing the variability in object appearance, intricate welding processes, restricted visibility, sensor interference, processing limitations, knowledge gaps, and safety considerations. Overcoming these hurdles requires the implementation of cutting-edge sensing and perception technologies, intricate software algorithms, and meticulous system integration. Within the realm of industrial robotics, welding robots grapple with various visual perception challenges. This encompasses current issues, potential solutions, and future prospects within the field of welding robotics. 

In the exploration of deep learning and convolutional neural networks (CNN) within the realm of robot welding vision systems, it is crucial to recognize the potential of alternative methodologies and assess their suitability in specific contexts. Beyond deep learning, traditional machine learning algorithms can be efficiently deployed in robot welding vision systems. Support vector machines (SVMs) and random forests, for example, emerge as viable choices for defect classification and detection in welding processes. These algorithms typically showcase a lower computational complexity and have the capacity to exhibit commendable performance on specific datasets. 

Rule-based systems can serve as cost-effective and interpretable alternatives for certain welding tasks. Leveraging predefined rules and logical reasoning, these systems process image data to make informed decisions. Traditional computer vision techniques, including thresholding, edge detection, and shape analysis, prove useful for the precise detection of weld seam positions and shapes. Besides CNNs, a multitude of classical computer vision techniques can find applications in robot welding vision systems. For instance, template matching can ensure the accurate identification and localization of weld seams, while optical flow methods facilitate motion detection during the welding process. These techniques often require less annotated data and can demonstrate robustness in specific scenarios. Hybrid models that amalgamate the strengths of different methodologies can provide comprehensive solutions. Integrating traditional computer vision techniques with deep learning allows for the utilization of deep learning-derived features for classification or detection tasks. Such hybrid models prove particularly valuable in environments with limited data availability or high interpretability requirements. 

The primary challenges encountered by robotic welding vision systems include the following:

1. Adaptation to changing environmental conditions: robotic welding vision systems often struggle to swiftly adjust to varying lighting, camera angles, and other environmental factors that impact the welding process.
2. Limited detection and recognition capabilities: conventional computer vision techniques used in these systems have restricted abilities to detect and recognize objects, causing errors during welding.
3. Vulnerability to noise and interference: robotic welding vision systems are prone to sensitivity issues concerning noise and interference, stemming from sources such as the welding process, robotic movement, and external factors like dust and smoke.
4. Challenges in depth estimation and 3D reconstruction: variations in material properties and welding techniques contribute to discrepancies in the welding process, leading to difficulties in accurately estimating depth and achieving precise 3D reconstruction.
5. The existing welding setup is intricately interconnected, often space-limited, and the integration of a multimodal sensor fusion system necessitates modifications to accommodate new demands. Effectively handling voluminous data and extracting pertinent information present challenges, requiring preprocessing and fusion algorithms. Integration entails comprehensive system integration and calibration, ensuring seamless hardware and software dialogue for the accuracy and reliability of data.

To tackle these challenges, the following solutions are proposed for consideration:

1. Develop deep learning for object detection and recognition: The integration of deep learning techniques, like convolutional neural networks (CNNs), can significantly enhance the detection and recognition capabilities of robotic welding vision systems. This empowers them to accurately identify objects and adapt to dynamic environmental conditions.
2. Transfer deep learning for welding robot adaptation: leveraging pre-trained deep learning models and customizing them to the specifics of robotic welding enables the vision system to learn and recognize welding-related objects and features, elevating its performance and resilience.
3. Develop multi-modal sensor fusion: The fusion of visual data from cameras with other sensors such as laser radar and ultrasonic sensors creates a more comprehensive understanding of the welding environment. This synthesis improves the accuracy and reliability of the vision system.
4. Integrate models and hardware: Utilizing diverse sensors to gather depth information and integrating this data into a welding-specific model enhances the precision of depth estimation and 3D reconstruction.
5. Perform a comprehensive requirements analysis and system evaluation in collaboration with welding experts to design a multi-modal sensor fusion architecture. Select appropriate algorithms for data extraction and fusion to ensure accurate and reliable results. Conduct data calibration and system integration, including hardware configuration and software interface design. Calibrate the sensors and assess the system performance to ensure stable and reliable welding operations.

Potential future advancements encompass the following:

1. Enhancing robustness in deep learning models: advancing deep learning models to withstand noise and interference will broaden the operational scope of robotic welding vision systems across diverse environmental conditions.
2. Infusing domain knowledge into deep learning models: integrating welding-specific expertise into deep learning models can elevate their performance and adaptability within robotic welding applications.
3. Real-time processing and feedback: developing mechanisms for real-time processing and feedback empowers robotic welding vision systems to promptly respond to welding environment changes, enhancing weld quality and consistency.
4. Autonomous welding systems: integrating deep learning with robotic welding vision systems paves the way for autonomous welding systems capable of executing complex welding tasks without human intervention.
5. Multi-modal fusion for robotic welding: merging visual and acoustic signals with welding process parameters can provide a comprehensive understanding of the welding process, enabling the robotic welding system to make more precise decisions and improve weld quality.
6. Establishing a welding knowledge base: creating a repository of diverse welding methods and materials enables robotic welding systems to learn and enhance their welding performance and adaptability from this knowledge base.

## 7. Conclusions

The rapid advancement of sensor intelligence and artificial intelligence has ushered in a new era where emerging technologies like deep learning, computer vision, and large language models are making significant inroads across various industries. Among these cutting-edge innovations, welding robot vision perception stands out as a cross-disciplinary technology, seamlessly blending welding, robotics, sensors, and computer vision. This integration offers fresh avenues for achieving the intelligence of welding robots, propelling this field into the forefront of technological progress.

A welding robot with advanced visual perception should have the following characteristics: accurate positioning and detection capabilities, fast response speed and real-time control, the ability to work in complex scenarios, the ability to cope with different welding materials, and a high degree of human–machine collaboration. Specifically, the visual perception system of the welding robot requires highly accurate image processing and positioning capabilities to accurately detect the position and shape of the welded joint. At the same time, the visual perception system needs to have fast image processing and analysis capabilities, which can perceive and judge the welding scene in real time in a short period of time and make correct control and feedback on abnormal situations in time. Actual welding is usually carried out in a complex environment, including interference factors such as lighting changes, smoke, and sparks. A good visually perceptive welding robot should have a strong ability to adapt to the environment and can achieve accurate recognition in complex environments. At the same time, the visual perception system of the welding robot needs to have the ability of multi-material welding and can adapt to the welding needs of different materials. Finally, with the development of smart factories, the visual perception system of welding robots needs to have the ability of human–computer interaction and collaboration.

At present, the most commonly used welding robot vision perception solution is based on the combination of vision sensor and deep learning model, through depth estimation and three-dimensional reconstruction methods to perceive the depth of the welding structure and obtain the three-dimensional information of the welding structure. Deep learning-based approaches typically use models such as convolutional neural networks (CNNS) to learn depth features in images. By training a large amount of image data, these networks learn the relationship between parallax, texture, edge, and other features in the image and depth. Through the image collected by the vision sensor, the depth estimation model can output the depth information of the corresponding spatial position of the image. This depth model may solve the problem that the welding robot needs to be accurately positioned in the space position, so that the attitude and motion trajectory of the welding robot can be controlled.

In conclusion, in the pursuit of research on robot welding vision systems, a balanced consideration of diverse methodologies is essential, with the selection of appropriate methods based on specific task requirements. While deep learning and CNNs wield immense power, their universal applicability is not guaranteed. Emerging or traditional methods may offer more cost-effective or interpretable solutions. Therefore, a comprehensive understanding of the strengths and limitations of different methodologies is imperative, and a holistic approach should be adopted when considering their applications.

## Figures and Tables

**Table 3:** Approaches based on deep learning in the selected papers.

Year	Method	Description	References
2014	Voxel	A supervised coarse-to-fine deep learning network is proposed, consisting of two networks, for depth estimation.	[104]
2015	Voxel	A method is proposed to represent geometric 3D shapes as a probabilistic distribution of binary variables in a 3D voxel grid.	[105]
2016	Voxel	The proposed 3D-R2N2 model utilizes an Encoder-3DLSTM-Decoder network architecture to establish a mapping from 2D images to 3D voxel models, enabling voxel-based single-view/multi-view 3D reconstruction.	[106]
2016	Voxel	Predicting voxels from 2D images and performing 3D model retrieval becomes feasible.	[107]
2016	Voxel	A novel encoder–decoder network is proposed, which incorporates a new projection loss defined by projection transformations.	[108]
2017	Point cloud	Exploring 3D geometric generation networks based on point cloud representations.	[109]
2018	Point cloud	A novel 3D generation model framework is proposed to effectively generate target shapes in the form of dense point clouds.	[110]
2019	Point cloud	A novel point cloud-based multi-view stereo network is proposed, which directly processes the target scene as a point cloud. This approach provides a more efficient representation, especially in high-resolution scenarios.	[111]
2023	Point cloud	An attention-based deep sparse prior cascade multi-view stereo network is proposed for 3D reconstruction.	[112]
2019	Point cloud	This study proposes the use of a data-driven deep learning framework to automatically detect and classify building elements from point cloud scenes obtained through laser scanning.	[113]
2019	Point cloud	Three-dimenional LMNet is proposed as a latent embedding matching method for 3D reconstruction.	[114]
2023	Point cloud	A learning-based method called GeoUDF is proposed to address the long-standing and challenging problem of reconstructing discrete surfaces from sparse point clouds.	[115]
2018	Mesh	Using 2D supervision to perform gradient-based 3D mesh editing operations.	[116]
2018	Mesh	The state-of-the-art incremental manifold mesh algorithm proposed by Litvinov and Lhuillier has been improved and extended by Romanoni and Matteucci.	[117]
2019	Mesh	A passive translation-based method is proposed for single-view mesh reconstruction, which can generate high-quality meshes with complex topological structures from a single template mesh with zero genus.	[118]
2020	Mesh	Pose2Mesh is proposed as a novel system based on graph convolutional neural networks, which can directly estimate the 3D coordinates of human body mesh vertices from 2D human pose estimation.	[119]
2020	Mesh	By employing different mesh parameterizations, we can incorporate useful modeling priors such as smoothness or composition from primitives.	[120]
2021	Mesh	A novel end-to-end deep learning architecture is proposed that generates 3D shapes from a single color image. The architecture represents the 3D mesh in graph neural networks and generates accurate geometries using progressively deforming ellipsoids.	[121]
2021	Mesh	A deep learning method based on network self-priors is proposed to recover complete 3D models consisting of triangulated meshes and texture maps from colored 3D point clouds.	[122]

**Table 4:** Research on sensor technologies for welding robots in different industrial fields.

Year	Area	Key Technology	Description	References
2015	Shipyard	Human–robot interaction mobile welding robot	Human–machine interaction mobile welding robots successfully remotely produced welds.	[126]
1999	Shipyard	Ship welding robot system	A ship welding robot system was developed for welding process technology.	[127]
2017	Shipyard	Super flexible welding robot	A super flexible welding robot module with 9 degrees of freedom was developed.	[128]
2014	Shipyard	Welding vehicle and six-axis robotic arm	A new type of welding robot system was developed.	[129]
2017	Automobile	Multi-robot welding system	An extended formulation of the design and motion planning problems for a multi-robot welding system was proposed.	[130]
2021	Automobile	Robot-guided friction stir welding gun	A new type of robot-guided friction stir welding gun technology was developed.	[131]
2020	Automobile	Friction welding robot	A redundant 2UPR-2RPU parallel robotic system for friction stir welding was proposed.	[132]
2010	Automobile	Arc welding robot	A motion navigation method based on feature mapping in a simulated environment was proposed. The method includes initial position guidance and weld seam tracking.	[133]
2017	Machinery	Visual system calibration program	A visual system’s calibration program was proposed and the position relationship between the camera and the robot was obtained.	[134]
2010	Machinery	Robot system for welding seawater desalination pipes	A robotic system for welding and cutting seawater desalination pipes was introduced.	[135]
2021	Aerospace	Aerospace friction stir welding robot	By analyzing the system composition and configuration of the robot, the loading conditions of the robot’s arm during the welding process were accurately simulated, and the simulation results were used for strength and fatigue checks.	[136]
2021	Aerospace	New type of friction stir welding robot	An iterative closest point algorithm was used to plan the welding trajectory for the most complex petal welding conditions.	[137]
2010	Aerospace	Industrial robot	Using industrial robots for the friction stir welding (FSW) of metal structures, with a focus on the assembly of aircraft parts made of aluminum alloy.	[138]
2014	Railway	Industrial robot	The system was developed and implemented based on a three-axis motion device and a visual system composed of a camera, a laser head, and a band-pass filter.	[139]
2017	Railway	Rail welding path grinding robot	A method for measuring and reconstructing a steel rail welding model was proposed.	[140]
2000	Railway	Industrial robot	Automation in welding production for manufacturing railroad car bodies was introduced, involving friction stir welding, laser welding, and other advanced welding techniques.	[141]
2018	Nuclear	New type of underwater welding robot	An underwater robot for the underwater welding of cracks in nuclear power plants and other underwater scenarios was developed.	[142]
2017	Nuclear	Robot TIG welding	Manual and robotic TIG welding used in key nuclear industry manufacturing was compared.	[143]
2020	PCB	Flexible PCB welding robot	A deep learning-based automatic welding operation scheme for flexible PCBs was proposed.	[144]
2023	PCB	Soldering robot	The optimized PCB welding sequence was crucial for improving the welding speed and safety of robots.	[145]
1997	Construction	Steel frame structure welding robot	Two welding robot systems were developed to rationalize the welding of steel frame structures.	[146]
2020	Construction	Steel frame structure welding robot	The adaptive tool path of the robot system enabled the robot to generate welds at complex approach angles, thereby increasing the potential of the process.	[147]
2020	Medical equipment	Surgical robot performing remote welding	The various challenges of using surgical robots equipped with digital cameras for remote welding, used to observe welding areas, especially the difficulty of detecting weld pool boundaries, were described.	[148]
2020	Medical equipment	Intelligent welding system for human soft tissue	By combining manual welding machines with automatic welding systems, intelligent welding systems for human soft tissue welding could be developed in medicine.	[149]

## Data Availability

Data are available upon reasonable request.
